# Effect of a Speaking Valve on Nasal Airflow During Tracheostomy Weaning: A Case Series

**DOI:** 10.1007/s12028-024-01966-8

**Published:** 2024-03-20

**Authors:** Thomas Gallice, Emmanuelle Cugy, Didier Cugy, Julie Laimay, Olivier Branchard, Christine Germain, Patrick Dehail, Emmanuel Cuny, Julien Engelhardt

**Affiliations:** 1 Aging, Chronic Diseases, Technology, Disability, and Environment Team, Bordeaux Research Center for Population Health, Bordeaux Segalen University, UMR_S 1219, 33000 Bordeaux, France; 2 Swallowing Evaluation Unit, Physical and Rehabilitation Medicine Unit, Tastet-Girard Hospital, Bordeaux University Hospital, 33000 Bordeaux, France; 3 Neurosurgery Unit B, Pellegrin Hospital, Bordeaux University Hospital, 33000 Bordeaux, France; 4 Neurological Intensive Care Unit, Pellegrin Hospital, Bordeaux University Hospital, 33000 Bordeaux, France; 5 Physical and Rehabilitation Medicine Unit, Arcachon Hospital, 33260 La Teste de Buch, France; 6 Physical and Rehabilitation Medicine Unit, Tastet-Girard Hospital, Bordeaux University Hospital, 33000 Bordeaux, France; 7 Sleep Medicine Unit, Pellegrin Hospital, Bordeaux University Hospital, 33000 Bordeaux, France; 8 Medical Information Unit, Pellegrin Hospital, Bordeaux University Hospital, 33000 Bordeaux, France; 9 Neurodegenerative Diseases Institute, Bordeaux University, 33000 Bordeaux, France; 10 CNRS, Neurodegenerative Diseases Institute, 33000 Bordeaux, France; 11 Polytechnic Institute of Bordeaux, Centre National de la Recherche Scientifique, Institut de Mathématiques de Bordeaux, Bordeaux University, 33400 Bordeaux, France

In the intensive care unit (ICU), many patients with acquired brain injury (ABI) benefit from tracheostomy [1]. Tracheostomy weaning protocols typically include cuff deflation and tube capping [2–4]. However, the roles and importance of these steps are debated. The rationale behind tube capping is to recreate airflow through the upper airway that promotes laryngeal reafferentiation, natural heating, air filtration, humidification through the nose, swallowing, and improved subglottic pressure [5, 6] (Fig. 1). However, tube capping can increase the respiratory workload by reducing the tracheal lumen diameter because it forces the airflow around the cannula [7] (Fig. 1, step 2 and b). Therefore, this may be considered risky or too demanding [7]. Cuff deflation without tube capping is sometimes suggested instead of cuff deflation with tube capping [8], but it has not been proven that this sufficiently recreates the upper airway airflow necessary for tracheostomy weaning.

**Fig. 1:**
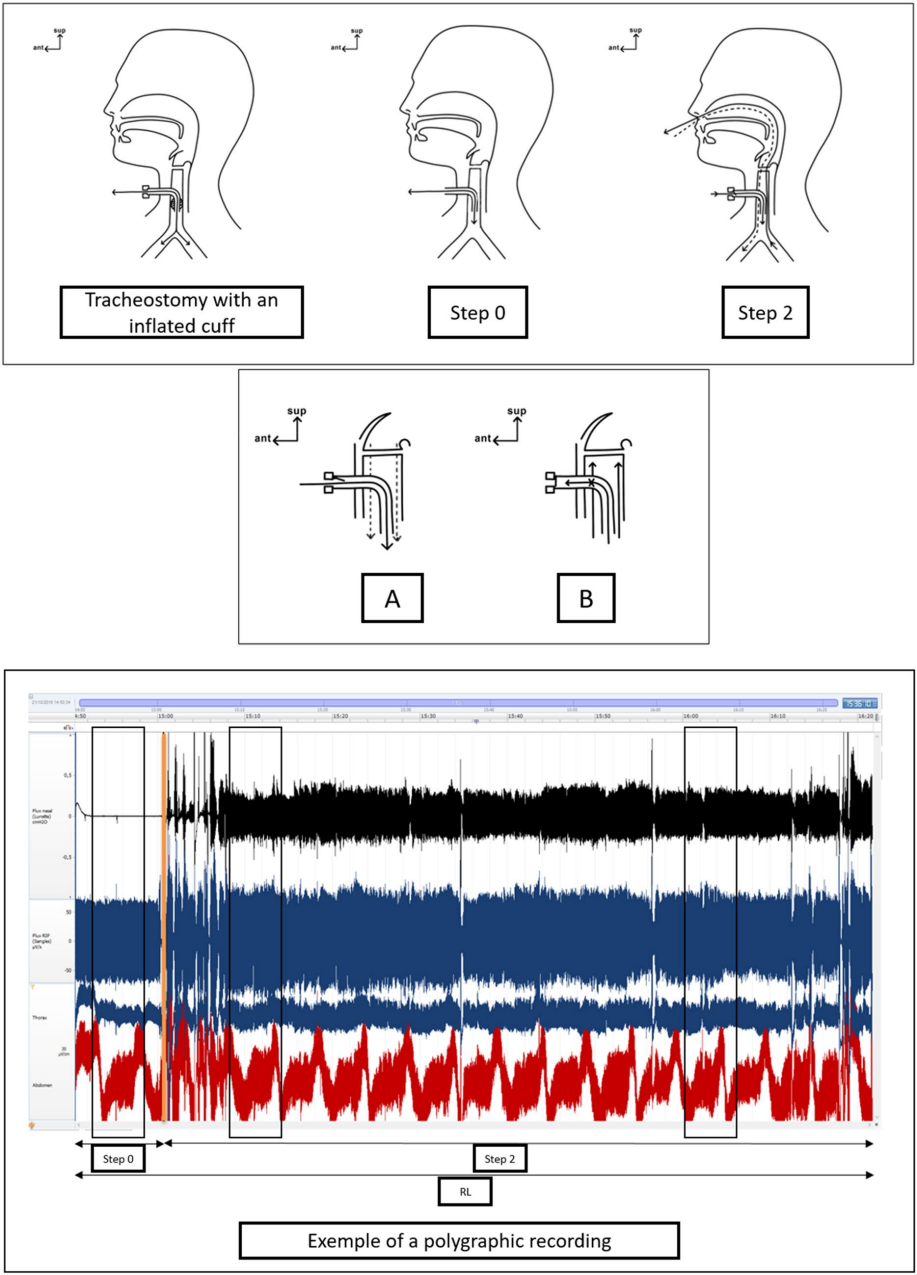
Upper panel: Tracheostomy with an inflated cuff. Airflow is possible only through the cannula (solid lines). Step 0, Airflow with a deflated cuff alone; solid lines: principal airflow routes (inspiratory and expiratory airflows through the cannula). Step 2, Airflow with a deflated cuff and a speaking valve; solid lines: the principal airflow routes (inspiratory airflow through the cannula, expiratory airflow through the nose); dashed lines: accessory airflow route (inspiratory airflow through the nose). Intermediate panel: An enlarged view of a tracheostomy tube inserted in the trachea. **a** The function of a speaking valve during inspiration; solid line, inspiratory airflow opens the valve and the air then goes through the cannula; dashed line, accessory inspiratory airflow through the upper airway. **b** The function of a speaking valve during expiration; expiratory airflow closes the valve and the air is then forced to pass through the upper airway. Lower panel: Example polygraph traces (patient 10): upper graph, nasal airflow; intermediate graph, respiratory inductance plethysmographic flow (RIP), and thus the reconstituted respiratory flow is based on the thoracic and abdominal movements; lower graph, the thoracic and abdominal movements. Step 0, Recording with a deflated cuff alone. Step 2, Recording with a deflated cuff and a speaking valve. Analyses employed clean 5-min samples at step 0, at the beginning of step 2, and 1 h thereafter if the patient remained in step 2 (black boxes). Vertical orange line, time of speaking valve placement. ant, anterior, RL, recording length (90 min), sup, superior

The objective of this study was to describe and compare the upper airway airflow, swallowing, and signs of increased respiratory workload under the following conditions: cuff deflation alone and cuff deflation with a speaking valve. To our knowledge, this comparison is novel.

This is a prospective case series (ClinicalTrials.gov NCT03512054) approved by ethics committee (Approval No. 17-12-08).

The inclusion criteria were age ≥ 18 years, hospitalization with ABI, tracheostomy performed in the ICU, weaned from mechanical ventilation (MV), scheduled for tracheostomy weaning, written informed consent from the patient or a legal representative, and patient access to the benefits of the French health care system.

The exclusion criterion was severe malnutrition defined as follows: body mass index < 16 kg/m^2^ or albuminemia < 20 g/L for patients aged < 70 years; body mass index < 18 kg/m^2^ or albuminemia < 30 g/L for patients aged > 70 years.

All patients underwent tracheostomy weaning following a five-step logigram: step 0, deflation of the tracheostomy cuff; step 1, brief manual occlusion of the cannula (a few seconds, 1 min maximum) to assess airway patency before speaking valve placement; step 2, placement of a speaking valve for a full 12 h; step 3, capping the cannula with a plug for a full 24 h; and step 4, decannulation (see Gallice et al. [9] for a full description of the protocol).

We compared the status of nasal airflow, swallowing, and the use of accessory respiratory muscles at step 0 (deflated cuff alone), the beginning of step 2 (deflated cuff with placement of a speaking valve), and 1 h after step 2. For each patient, we used polygraphy to continuously record the nasal respiratory flows (inspiratory and expiratory) and thoracic and abdominal movements during the first 2 h of tracheostomy weaning. Over this period, we expected that patients would complete steps 0 and 1 and that step 2 would commence and last at least 1 h after the start of the weaning process. We used a ResMed Nox Polygraph (PG) (San Diego, CA). We recorded nasal airflow using a nasal cannula. Each recording was obtained with the mouth closed to avoid signal loss associated with mouth breathing. We recorded thoracic and abdominal movements using thoracic and abdominal straps (Fig. 1, lower panel). We evaluated breathing status based on the movements of the abdominal and thoracic straps shown on the PG recording. Data were extracted from the PG and anonymized in.edf format using ResMed Noxturnal software. Three stages of the entire recording were analyzed: step 0, the beginning of step 2, and 1 h thereafter if the patient remained in this step (step 2 plus 1 h) (Fig. 1, lower panel). Cuff deflation and the use of a speaking valve can cause intense coughing, which may affect the quality of data recording. Therefore, for each patient, we selected a clean sample of 5 min for each period for analysis. If it was impossible to obtain a clean 5-min sample, we selected a clean sample of the maximum possible duration. The data-processing module was specifically developed (by one of the authors, DC) for this study using the Xojo 2018 Release 2 computer-based tool integrated with signal display PGS-OSX software.

Activation of accessory inspiratory muscles (qualitative results: yes or no) at step 0 and step 2 was clinically observed and recorded by the physiotherapist who was implementing the protocol. These data served as indirect assessments of respiratory workload.

The numbers of spontaneous swallows were recorded at step 0 and step 2. A swallow was defined as a complete elevation of the larynx observed by the physiotherapist implementing the protocol. Observations were made during the first 5 min of steps 0 and 2. We also collected data on age, sex, tracheostomy model, Coma Recovery Scale revised score at inclusion, type of ABI, ICU length of stay, MV duration, time from intubation to tracheostomy weaning, functional status at ICU discharge, and lesion location (supratentorial, infratentorial, or both).

Statistical analysis employed SAS software (version 9.4; Cary, NC), with the two-sided type I error rate set to 0.05. The baseline quantitative patient characteristics are presented as numbers with means and standard deviations (SDs), or as medians with interquartile ranges (IQRs). Nasal airflows are given as means with SDs or as medians with minimum and maximum values. Given the characteristics of the sensor, the airflows were estimated using the pressures recorded by the nasal cannula and are thus expressed in cmH_2_O. Airflows were compared between steps 0 and 2, steps 0 and 2 plus 1 h, and steps 2 and 2 plus 1 h using the Wilcoxon signed-rank test. The numbers of swallows at steps 0 and 2 were compared in the same manner.

Our results were as follows: after discharge from neurological or traumatic ICUs to two neurosurgery units, all of 15 patients (10 women) were consecutively and exhaustively enrolled from 27/05/2019 to 21/12/2019. Three patients (patients 4, 8, and 15) failed to pass step 1 (manual tube occlusion with a deflated cuff) or did not tolerate the procedure. They exhibited airway patency impairments incompatible with appropriate analysis of nasal airflow. Therefore, they were excluded from statistical analysis. One patient passed step 2 but failed to continue to step 2 plus 1 h (patient 14). For the 12 patients included in analysis, the median age was 53 years (IQR 40–60 years); the median Coma Recovery Scale revised was 18 (IQR 13–21); the mean time from intubation to tracheostomy weaning was 57 days (SD 26 days); the mean ICU length of stay was 41 days (SD 15 days); and the mean MV duration 28 h (SD 10 h). Eight patients had supratentorial lesions, five had infratentorial lesions, and two had both (Table 1).

**Table 1:** Patient characteristics

Patient	Age	Sex	CRS-r	ICU LOS (d)	MV duration (d)	Time from intubation to TW (d)	Type of ABI	Functional status at ICU discharge	TW protocol	Model of tracheostomy
1	32	M	23	43	40	62	Fourth ventricle hemangioblastoma	mRS 4, cerebellar ataxia	Passed	Rusch 10
2	52	M	17	41	36	48	SAH (basilar trunk aneurysm)	mRS 5	Passed	Rusch 10
3	70	M	22	30	27	64	Left pontocerebellar angle meningioma	mRS 5, right hemiplegia	Passed	Shiley 6LPC
4	36	F	19	30	16	38	ICH, SAH (left sylvian aneurysm)	mRS 5, right hemiplegia	Failed at step 1	Shiley 6LPC
5	47	F	3	20	13	26	SAH (right internal carotid aneurysm)	mRS 5, left hemiplegia	Passed	Rusch 8.5
6	56	F	22	40	27	50	Severe TBI	mRS 4, left hemiplegia	Passed	Rusch 8.5
7	32	M	6	62	38	85	SAH (right sylvian aneurysm)	mRS 5	Passed	Rusch 8.5
8	65	F	23	36	26	56	SAH (anterior communicating artery aneurysm)	mRS 4, right hemiplegia	Failed at step 1	Rusch 8.5
9	27	F	20	74	38	115	Left ICH	mRS 5	Passed	Rusch 8.5
10	63	F	18	38	11	50	SAH (right sylvian aneurysm)	mRS 5, left hemiplegia	Passed	Rusch 8.5
11	61	F	16	38	36	47	SAH (right internal carotid aneurysm)	mRS 5	Passed	Rusch 8.5
12	46	F	19	34	28	62	Posterior fossa astrocytoma	mRS 5, left hemiplegia	Passed	Shiley 6LPC
13	59	F	14	47	26	56	SAH (pericallosa artery aneurysm)	mRS 5	Passed	Rusch 8.5
14	53	F	12	22	17	23	SAH (right internal carotid aneurysm)	mRS 5, left hemiplegia	Failed at step 2 + 1 h	Rusch 8.5
15	40	M	23	21	12	22	Superior vermian ICH	mRS 4, cerebellar ataxia	Failed at step 1	Rusch 8.5

*ABI* Acquired brain injury, *CRS-r* Coma recovery scale, revised, *F* Female, *ICH* Intracerebral hemorrhage, *ICU* Intensive care unit, *LOS* Length of stay, *M* Male, *mRS* Modified Rankin Score, *MV* Mechanical ventilation, *SAH* Subarachnoid hemorrhage, *TBI* Traumatic brain injury, *TW* Tracheostomy weaning

All patients had been tracheostomized in the ICUs using a percutaneous technique. We used the following tracheostomy tubes: Rusch size 8.5 for ten patients (internal diameter [ID] 8.7 mm, outer diameter [OD] 10.3 mm), Rusch size 10 for two patients (ID 10.2 mm, OD 12.3 mm), and Shiley 6LPC for three patients (ID 6.4 mm, OD 10.8 mm). When performing tube capping, we used Shiley speaking valves exclusively. Prigent et al. [10] demonstrated that the work of breathing was higher when the Rusch speaking valve rather than when the Shiley valve was used.

The complete results are presented in Table 2.

**Table 2:** Nasal airflows at step 0, step 2, and step 2 + 1 h and the numbers of swallows at step 0 and step 2

Variable	Step 0	Step 2	Step 2 + 1 h	Differences per patients	*P* value
Nasal airflow (step 0 vs. step 2) (cmH_2_O)					
*n*	12	12			0.0049*
Mean (SD)	0.25 (0.24)	0.82 (0.71)		0.57 (0.64)	
Median (Q1–Q3)	0.19 (0.06–0.38)	0.51 (0.28–1.29)		0.32 (0.10–1.10)	
Min, max	0.01, 0.80	0.18, 2.23		− 0.21, 1.74	
Nasal airflow (step 0 vs. step 2 + 1 h) (cmH_2_O)					
*n* (m.d.)	12		11(1)		0.0293*
Mean (SD)	0.25 (0.24)		0.66 (0.61)	0.41 (0.59)	
Median (Q1 to Q3)	0.19 (0.06 to 0.38)		0.35 (0.19 to 1.14)	0.11 (0.00 to 0.86)	
Min, max	0.01, 0.80		0.01, 1.91	− 0.45, 1.58	
Nasal airflow (step 2 vs. step 2 + 1 h) (cmH_2_O)		12	11(1)		0.2324
*n* (m.d.)		12	11(1)		0.2324
Mean (SD)		0.82 (0.71)	0.66 (0.61)	− 0.16 (0.46)	
Median (Q1 to Q3)		0.51 (0.28 to 1.29)	0.35 (0.19 to 1.14)	− 0.06 (− 0.42 to 0.00)	
Min, max		0.18, 2.23	0.01, 1.91	0.01, 1.91	
Number of swallowing/5 min					
*n*	12	12			0.2715
Mean (SD)	2.5 (2.4)	3.7 (2.8)		1.17 (3.04)	
Median (Q1 to Q3)	2 (1 to 5)	4 (2 to 5)		1.50 (− 0.50 to 3.00)	
Min, max	0, 7	0, 10		− 4.00, 7.00	

The *p* values are for the Wilcoxon signed-rank test, * bold values are for p values < 0.05

Max, maximum, m.d., missing data, min, minimum, Q1, quartile 1, Q3, quartile 3, SD, standard deviation

No involvement of accessory respiratory muscles was recorded at step 0 or step 2.

From our findings, a speaking valve with a deflated cuff significantly enabled nasal respiratory airflow without the involvement of accessory respiratory muscles compared with a deflated cuff alone (Table 2, Fig. 1). However, we did not find a significant difference in the numbers of swallows between the two conditions (despite a tendency toward a higher number of swallows when a speaking valve was placed; see Table 2). Conversely, in an earlier study, Kim et al. [11] found qualitative improvement of swallowing function with the use of a speaking valve; however, the number of swallows was not included in the study results.

The difference in nasal airflow might be explained by the fact that, without a speaking valve, the shortest and easiest route for the airflow would likely be through the cannula. Chadda et al. [12] reported that the dead space above the cannula accounted for 30% of the workload. Therefore, if tube capping is not used to force airflow through the upper airway, the patient may breathe only through the cannula, even with a deflated cuff. This is in line with the findings of Prigent et al. [13], who found that expiratory flow after swallowing was present when a speaking valve was placed but was negligible without a valve. The presence of an inspiratory nasal airflow with a speaking valve is surprising because, theoretically, inspiratory airflow would be expected solely through the tube [14] (Fig. 1, step 2, a, b). The use of a speaking valve likely increases the respiratory workload to a level that allows inspiratory airflow around the cannula, and then through the upper airway, but not enough to engage the accessory respiratory muscles. Further studies are needed to confirm this hypothesis. Moreover, we found that this effect was maintained at 1 h (step 2 plus 1 h), proving that it is not simply a brief effect of speaking valve placement.

The major limitation of this study was the small sample size, which precluded us from drawing firm conclusions. However, to our knowledge, this is the first time that nasal airflow has been recorded during tracheostomy weaning of patients with ABI. The PG used to record nasal airflow could be replaced by a pneumotachograph [12]. Instrumental assessments and esophageal pressures would aid swallowing and respiratory workload assessments [15, 16]. Such examinations require that patients are moved out of their units, invasive devices are used, and patients can cooperate; additionally, they cannot be performed simultaneously. Thus, such assessments are at least difficult and may be dangerous when tracheotomy weaning is initiated.

To conclude, the use of a speaking valve with a deflated cuff restores airflow through the upper airway more effectively than cuff deflation alone (Fig. 1, step 0, step 2). A speaking valve restores not only the expiratory nasal airflow but also, to a lesser degree, the inspiratory nasal airflow (Fig. 1). Hence, because restoring airflow in the upper airway is considered key during swallowing rehabilitation of tracheostomized patients, our findings seem to favor the use of a speaking valve rather than cuff deflation alone during tracheostomy weaning.

## References

[1] Bösel J. Tracheostomy in stroke patients. Curr Treat Options Neurol janv. 2014;16(1):274. 10.1007/s11940-013-0274-1. 10.1007/s11940-013-0274-1 24357462

[2] Singh RK, Saran S, Baronia AK. The practice of tracheostomy decannulation—a systematic review. J Intensive Care. 2017;5(1):38. 10.1186/s40560-017-0234-z. 28649385 10.1186/s40560-017-0234-z PMC5477679

[3] Pandian V, Miller CR, Schiavi AJ, Yarmus L, Contractor A, Haut ER, et al. Utilization of a standardized tracheostomy capping and decannulation protocol to improve patient safety: tracheostomy capping and decannulation protocol. Laryngoscope. 2014;124(8):1794–800. 10.1002/lary.24625. 24473939 10.1002/lary.24625

[4] Hess DR. Facilitating speech in the patient with a tracheostomy. Respir Care. 2005;50(4):7. 15807915

[5] Robert D. Les troubles de la déglutition postintubation et trachéotomie. Réanimation. 2004;13(6–7):417–30. 10.1016/j.reaurg.2004.06.002.

[6] Suiter DM, McCullough GH, Powell PW. Effects of cuff deflation and one-way tracheostomy speaking valve placement on swallow physiology. Dysphagia. 2003;18(4):284–92. 10.1007/s00455-003-0022-x. 14571334 10.1007/s00455-003-0022-x

[7] Hernández Martínez G, Rodriguez ML, Vaquero MC, Ortiz R, Masclans JR, Roca O, et al. High-flow oxygen with capping or suctioning for tracheostomy decannulation. N Engl J Med. 2020;383(11):1009–17. 10.1056/NEJMoa2010834. 32905673 10.1056/NEJMoa2010834

[8] Trouillet JL, Collange O, Belafia F, Blot F, Capellier G, Cesareo E, et al. Trachéotomie en réanimation. Anesth Réanim. 2018;4(6):508–22. 10.1016/j.anrea.2018.08.003.

[9] Gallice T, Cugy E, Germain C, Barthélemy C, Laimay J, Gaube J, et al. A pluridisciplinary tracheostomy weaning protocol for brain-injured patients, outside of the intensive care unit and without instrumental assessment: results of pilot study. Dysphagia. 2023. 10.1007/s00455-023-10641-7. 38062168 10.1007/s00455-023-10641-7 PMC11239749

[10] Prigent H, Orlikowski D, Blumen MB, Leroux K, Legrand L, Lejaille M, et al. Characteristics of tracheostomy phonation valves. Eur Respir J mai. 2006;27(5):992–6. 10.1183/09031936.06.00009405 16707394

[11] Kyun KY, Heon LS, Won LJ. Effects of capping of the tracheostomy tube in stroke patients with dysphagia. Ann Rehabil Med. 2017;41(3):426–33. 10.5535/arm.2017.41.3.426. 28758080 10.5535/arm.2017.41.3.426 PMC5532348

[12] Chadda K, Louis B, Benaïssa L, Annane D, Gajdos P, Raphaël J, et al. Physiological effects of decannulation in tracheostomized patients. Intensive Care Med. 2002;28(12):1761–7. 10.1007/s00134-002-1545-6. 12447520 10.1007/s00134-002-1545-6

[13] Prigent H, Lejaille M, Terzi N, Annane D, Figere M, Orlikowski D, et al. Effect of a tracheostomy speaking valve on breathing–swallowing interaction. Intensive Care Med. 2012;38(1):85–90. 10.1007/s00134-011-2417-8. 22113817 10.1007/s00134-011-2417-8

[14] Passy V. Passy-muir tracheostomy speaking valve. Otolaryngol Head Neck Surg. 1986;95(2):247–8. 10.1177/019459988609500224. 3108769 10.1177/019459988609500224

[15] Dziewas R, Allescher HD, Aroyo I, Bartolome G, Beilenhoff U, Bohlender J, et al. Diagnosis and treatment of neurogenic dysphagia – S1 guideline of the German society of neurology. Neurol Res Pract. 2021;3(1):23. 10.1186/s42466-021-00122-3. 33941289 10.1186/s42466-021-00122-3 PMC8094546

[16] Bellani G, Pesenti A. Assessing effort and work of breathing. Curr Opin Crit Care. 2014;20(3):352–8. 10.1097/MCC.0000000000000089. 24722059 10.1097/MCC.0000000000000089

